# Parks and Tourism

**DOI:** 10.1371/journal.pbio.1000143

**Published:** 2009-06-30

**Authors:** Ralf Buckley

**Affiliations:** International Centre for Ecotourism Research, Griffith University, Southport, Queensland, Australia

## Abstract

To protect biodiversity, parks agencies need political and financial support. Recreational visitors can bring both, but commercial tourism carries risks.

Why should it matter how many people visit national parks? In a word: politics. Protected areas are not only physical places, reservoirs of biodiversity, and sources of ecosystem services, such as breathable air and drinkable water. They are also human political constructs, and they are under ever-increasing pressures from growing human populations and resource demands. Visitors may bring them the political capital to survive.

Biologists have pointed out for decades that protected areas are not playgrounds, but life-support systems for the planet's population of humans, as well as its other species. Economists estimate that ecosystem services worldwide contribute twice as much to the human economy each year as all forms of human industry combined—many trillions of dollars [1],[2]. At regional scale, ecosystem services from National Wildlife Refuges in the contiguous 48 states of the Unites States have been valued at US$27 billion annually [3]. The human economic value of conserving biodiversity is many orders of magnitude higher than the funds invested in it [2],[4]–[9]. The cost of buying all of the world's biodiversity hotspots outright has been estimated at around US$100 billion—less than five-years' expenditure on soft drinks in the US [10]. But the actual funds allocated worldwide each year, a few billion dollars in total, are <5% of minimum requirements for effective conservation [11]. This compares with the trillions of dollars spent in 2009 to prop up financial systems in the US, European Union, and China [12].

Both ecological and economic arguments thus support conservation investment orders of magnitude higher than those currently in place. Public conservation decisions, however, are political, and the currency of politics is power. Votes, money, or force can buy political power in various circumstances, but demonstrating that conservation has a high global ecological or economic value does not generate political capital—even in democracies—unless voters in marginal electorates will change their voting preferences on this issue above all others. Multi-trillion dollar economic valuations influence political processes only indirectly.

Conservation in the real world [13] relies on coupled social–ecological systems [9]. Visitors in parks provide an excellent example. Park managers must juggle ecological impacts and political support. Visitors create costs for conservation through ecological impacts [14] and by diverting conservation budgets to recreation management. Costs depend on numbers, timing, activities, equipment, and behaviour.

Visitors can also provide funds from entry and activity fees, as well as political capital, which buys government budget allocations. That's why parks agencies court recreational visitors through “relationship marketing” [15]. Government powerbrokers see park visitors as political supporters and regional spenders. More people care about conservation than recreation, but recreation means countable votes in specific electorates, whereas conservation concerns are less localised or vote-changing.

The tourism industry is also a powerful political player that sees parks as commercial opportunities—scenic attractions, captive clients, and publicly funded infrastructure, interpretation, and marketing. Commercial nature and adventure tourism is indeed growing, but building commercial accommodation inside parks does not increase visitation. Some commercial tourism operations do run profitable private reserves that make net positive contributions to conservation [16],[17], but these are much smaller than public-protected area systems and conservation stewardship schemes [18],[19].

Some parks agencies earn up to 80% of total revenue by charging individual visitors directly. Partnerships with tourism developers, however, have incurred high costs, brought few visitors and minimal revenue (<6%), earned no net revenue for conservation, and reduced benefits for private recreational visitors [20]. This approach also brings risks. If individual visitors cause impacts, agencies can restrict access or activities. This is not politically feasible for large private developments. Arguments advanced by commercial tourism interests are not supported by evidence; however, this is lobbying, not logic.

So if fewer people visit parks, it creates political problems for conservation. Historically, park managers worried about crowding, with conflicts and impacts, and about demands from people of different ethnic origins and socioeconomic backgrounds [21]. In the early 2000s, however, US researchers Pergams and Zaradic [22] argued that US society is experiencing “videophilia,” a preference for virtual reality over nature. Their data indicated declines in visitation to national parks in Japan and the US and, to a lesser extent, also other US land tenures since about 1990. The political implications of this led to intense debate [23]–[25].

The article by Balmford et al. [26], in this issue of *PloS Biology*, presents a large new set of data in this debate. They obtained visitation data for 280 protected areas in 20 countries worldwide, a much broader dataset geographically than that of Pergams and Zaradic [22]. Balmford et al. report that in most countries, park visitation rates are continuing to rise. In the practical politics of global conservation, these are critically important and timely data. The authors' research also showed, however, that in a few particular parks in the US numbers have remained static. So, the debate will continue, especially given the money at stake for the commercial tourism sector.

There are two key issues. First, few countries have accurate visitor numbers. Reliable counts need continuously staffed access roads with no other entry. Automated counters are expensive and inaccurate. Visitor numbers vary daily by orders of magnitude depending on weather and holiday periods, so comparing single-day snapshot counts means little. Calculating long-term trends needs continuous multi-year time series. These are rare, especially since land management agencies often change the basis for recording visits.

Pergams and Zaradic [22], for example, also sought data from countries such as Australia. They were sent data from two of eight states, one of which did not actually record visitor numbers—the figures were purely estimates. Australian tourism lobbyists quote park visitation estimates derived from very general off-site surveys of people's holiday intentions, carried out by the federal tourism agency. Such surveys are highly unreliable [22]. Even in face-to-face interviews with people who know you have been watching them, many report their own very recent actions inaccurately [27]. On-ground counts show the tourism surveys are inflated by 20–1,000% [28]. So, it's hard to measure small changes reliably.

Second, if visitation to particular parks has levelled off, this does not mean that parks are unpopular. It means that these parks are full. Their social carrying capacity [29] has been exceeded. They are so crowded that people go to other parks or other land tenures. Pergams and Zaradic [22] argued against such substitution within the US, but their data, especially for the US Forest Service, were not entirely convincing. Balmford et al. [26] propose instead that people have been travelling overseas; international outdoor tourism has been substituting for domestic outdoor recreation. This is supported by tourism research [17]. Crowding is not the only factor. Reduced public funding forces parks agencies to charge higher visitor fees, and increased legal liabilities make them impose more regulations. So people go where they enjoy cheaper and less fettered recreational opportunities.

And why not? It is good policy for people to play where their impact causes less damage, keeping protected areas for conservation and low-impact individual recreation. The former gives the human economy its air, water, and biological resources; the latter gives the human population low-cost improvements in physical and mental health. It's poor policy that in order to maintain budgets, parks agencies should continually increase visitor numbers. Human populations are growing, but the area of parks is not keeping pace. The area of land and water available for conservation outside protected areas is continually shrinking [30], so parks themselves are increasingly critical. Parks are assets for tourism, but they are not tourism assets.

The new data from Balmford et al. [26] show that park visitation rates are still rising. Conservation is a far more valuable use of parks than tourism and recreation, so in theory, parks agency budgets should only reflect conservation management costs, and visitation rates should be irrelevant. In practice, however, since conservation decisions are political rather than economic, these new data are of enormous importance to conservation worldwide.
